# Interview: Maria‐Carla Saleh

**DOI:** 10.1111/cmi.13061

**Published:** 2019-06-17

**Authors:** Maria‐Carla Saleh

**Affiliations:** ^1^ Virology Institut Pasteur Paris France

1

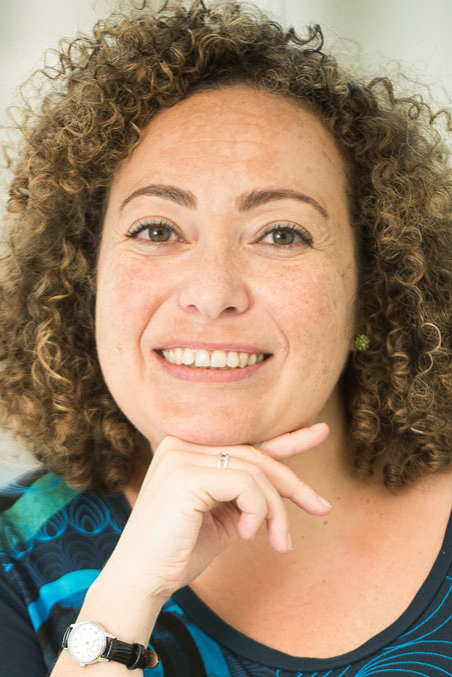

## 2 AUTHOR BIOGRAPHY

**Maria Carla Saleh** was born in Argentina, where she finished her Masters' degree in Biology at the National University of Cordoba. She obtained her PhD on Cellular and Molecular Physiopathology at the University of Paris 6 and then went to United States, where she worked as a postdoc at UCSF focusing on insect antiviral immunity. In 2008, she secured a position as a junior group leader at the Institut Pasteur of Paris and was tenured in 2013. Carla Saleh's research dissects the immune system of insects to answer how they remain asymptomatic while being infected with viruses that are deadly when transmitted to humans and how to manipulate this immune system to prevent transmission of viruses to humans from insect bites. With her team, she develops a unique combination of basic science and bioinformatics that allow them to tackle several projects in ill‐studied areas of insect antiviral immunity, host–pathogen biology, and emerging viral disease transmission.

### 2.1 What is your research background—what did you first start working on at the beginning of your academic career?

I am a biologist by diploma and a scientist by choice. I obtained my degree at the National University of Córdoba, Argentina. I also obtained the degree of Professor in Biology that allows teaching Biology in schools. For this second diploma, in addition to biology, I studied the conceptual and methodological frameworks of pedagogy and didactics and their application to the study of Biology according to different learning theories.

My first job as a scientist was during the summer of 1990. I worked in the laboratory of Histology and Cellular and Molecular Biology. We learned to critically read and present papers, and I learned the basis of histology: paraffin embedding of chicken embryos, cutting of sections, staining to visualise different tissues and cell types, cell morphology, etc. The year before obtaining my degree (1993), I was selected to perform a summer internship in Buenos Aires, at the Institute of Agriculture, and there I learned the basis of molecular biology: cloning, digestion with restriction enzymes, Southern blotting, etc. I was collaborating on a project that intended to clone and transform a tomato gene to render it resistant to hydric stress.

For my Masters (1994), I worked in a lab in the School of Chemistry. We optimised the molecular diagnosis of cystic fibrosis from blood and did an epidemiological study of the prevalence of cystic fibrosis mutations in the population of Córdoba. And I generated my first author paper! And then it became the “first moment of truth”: Do I want to do a PhD? I knew I wanted to keep exploring, learning, and discovering. So even if doing a PhD was not a “real job” to my family and friends, I obtained a fellowship from CONICET (the research national agency) and started my PhD working on transposable elements in cow brain. Why cows, you must wonder. Because obtaining fresh material from cows was easy and cheap those days in Argentina. I also recovered the brain of dead snakes and dead turtles in the zoo and we wrote a paper about the transposon we were studying with the postdoc that supervised me. By then, I was madly in love with a guy that obtained a postdoctoral fellowship to study in Paris and it was without much hesitation that I left everything behind and followed him to Paris. And… I started my PhD again, from scratch (year 1997). And now I was working in Institut Pasteur, with a C Marie Skłodowska‐Curie fellowship for PhD students. My thesis subject was the effect of transferrin produced by oligodendrocytes in the production and maintenance of myelin in the central nervous system. It seems I had set my career path to become a neuroscientist.

### 2.2 Have you always wanted to work in academia?

From the first time I set foot in a lab and tried to answer a question through research and scientific methodology, I knew I wanted to be “that person” that invents projects, pursues ideas, builds a team, and tries to discover. I did not know what an “academic career” meant, but I knew I wanted to do “that.” I had never met scientists before but I felt I could become one.

### 2.3 How has your early work brought you to what you are working on now?

As I mentioned above, I believed I was set to become a neuroscientist. And as you probably know, I am studying antiviral immunity in insects …. How did it happen? By the end of my PhD (and with the arrogance that youth gives) I was growing frustrated with the lack of advance in the field of neuroscience to find a cure for diseases such as Alzheimer's, MS, or ALS. I have always strongly felt that is our responsibility as scientists to assure that the prolonged life span that we have promoted through the discovery and cure of several diseases and ailments is accompanied by dignity and life quality. To forget who you are or who are the persons that you love the most, to forget your identity, your past and present, to forget your essence and what makes you human, have always been a cause of great suffering and distress for me. It is devastating for millions of patients and their families/friends around the globe, and science should address and fix it. As I said, I found that the progress being made was too slow and I decided to study immunology and virology to design a therapy that will invade the brain with minimal immune activation and would revert the disease. Yes, you can laugh at my arrogance and my naiveté. I left Paris and joined the lab of Raul Andino, at UCSF, to study viruses in the brain. By this time (late 2001), RNAi was making the news and with Raul we conceived this crazy project to show that RNAi was the immune response against viruses in the brain of mammals. Yes, you can laugh again. I started this project even before Dicer and Argonaute were cloned and sequenced! After many years of failure, we decided to work in an organism where RNAi was proven to exist and this is how my love affair with Drosophila started. My postdoctoral work showed that RNAi was the major antiviral response in insects, that the siRNA pathway needed an intact endocytosis mechanism to function, and that RNAi was systemic, meaning that the RNAi antiviral response protected not only the infection site but the entire organism by means of transport of a (yet unknown) immune signal. By the time I finished my postdoc and applied for jobs in academia, I was totally committed to insect immunity and to develop novel strategies to stop viruses in insects before they are transmitted to humans. What happen to the brain? I have not had my last word yet. One day, I will close the circle and find a way to target the human brain with therapeutic signals carried by viruses or virus‐derived cellular organelles. I am building my path in science as I walk. And the road can take unexpected turns.

### 2.4 Have you always been interested in insect immunity?

I have always been interested in the acquisition of knowledge by understanding processes. I am an ardent defender of knowledge as a shield against obscurantism. I am an ardent defender of access to knowledge (and let me stress the difference between knowledge and information) for everybody. I am a scholar by nature, I love to study and to learn. Each subject I researched during my scientific path was done with the same intensity, passion, and conviction I am doing it today. I find in my current field the source of motivation, energy, intellectual, and societal challenge that allows me to keep going on with the subject, that boosts my conviction in science and scientific knowledge as a response for human ailments. Will this love for insect immunity last for the remaining of my scientific career? Probably. Always with the same intensity? Probably not. What is next then? I will know it when it hits me.

### 2.5 What do you think the most interesting questions are at the moment in insect immunity?

RNAi is a nucleic acid‐based immune system that keeps holding unsolved mysteries. How widespread in the tree of life is the antiviral activity of RNAi? What is the nature of the immune signal that is propagated? How is the immune signal moving? Do insects develop memory to previous encountered infections? Is this memory transmitted to the next generation? What are the molecular bases of this memory? Are the RNAi components permanently induced? Does a dsRNA receptor exist? Is the RNAi‐mediated antiviral response organ‐specific? How do the different RNAi pathways interact to control a viral infection in an insect? Can we successfully manipulate RNAi to control vector borne diseases and plant pests?

### 2.6 Where do you think insect immunity is headed in the future? What do we need to be thinking about?

I think we have to be open‐minded and be ready to be surprised. When we talk of antiviral immunity in invertebrates, we are only scratching the surface of a complex phenomenon. Nucleic acids (RNA and DNA), proteins, lipids, extracellular vesicles, reverse transcriptases, endonucleases, exonucleases are all dancing together. Imagine this scene! We recognise the dance, we do not recognise the music. Biological “déjà vu” and biological innovation. We need to be thinking at subcellular localizations of complexes at high resolution (with the help of new powerful microscopy and artificial intelligence), we have to study each natural pathosystem, we have to consider the impact of microbiome and virome during a given viral infection, we have to think of immunity at the insect population level, in the natural environment where the infection is happening. Somehow, we have to build scientific bridges where ecology meet the bench and vice versa.

### 2.7 What do you enjoy the most about your work? And what about the least?

I must say that, even if I complain, I love my work. Each facet of it. Sometimes I go to the gel imaging machine when people in the lab are developing a gel for a result we were waiting for. And I still feel all these butterflies in the stomach that I used to feel when I was a student and I silently prayed while the UV lights were turned on, this anticipation of what it could be. The same butterflies come when I lay eyes for first time in a sequencing result or a western blot …. Just outside my office is the printer of the lab and I confess that I always peek to the graphs being printed: all these histograms, gels, alignments, curves, and even better if statistics are present! Then my heart races and my mind starts flying.

And I also tremendously enjoy seeing the students and postdocs blossoming. They grow in front of my eyes, I enjoy this sparkle in their eyes when they arrive full of motivation, I feel proud when they are right and I am wrong. I love to stay late talking with them about life and science, and scientific life, and fulfilment on all aspects of life. I love when we draw crazy theories on napkins. I enjoy the interactions that help me become a better PI each day so I can help them get prepared to be the PIs and the scientists of the future. And even if sometimes (or frequently) there are frustrations, disappointments, relationships that turn difficult, struggles of communication, I care for this sense of family that we have. Because “this is us.”

What I enjoy the least? The hassle of getting published. Do not take me wrong: I love writing papers, I love the process of creating a manuscript and telling the story with students and postdocs. Even more important, I believe in the peer review system and reviewers had consistently made our manuscripts better, more solid, and clear. But each manuscript takes at least 18 months from the first submission to acceptance and the process is wearing. By the day the paper is finally accepted, students and postdocs are often gone, I hate the manuscript, and we have moved into the next chapter of our scientific adventures. And because this is the space to complain, I do not like to write grant reports every 6 months (not even every year), I do not like to apply for grants every year and spend countless hours in meetings. But hey, this is also part of the package. So I play imaginary games during boring meetings, I take notes of phrases that sound smart, and try to guess what each person will answer or who will answer last using every one idea and looking as the smartest person in the room;‐).

### 2.8 What has been the most surprising result you have had in your career so far?

This is a difficult question to answer! Academically, each manuscript reports a surprise. Personally, everyday I am still surprised I got here ….

### 2.9 What advice would you give to other early career researchers starting out in their career given your experiences?

Follow your instinct but do not think that you are infallible or invincible. Ask for help, do not be shy, do not be ashamed, because we all, permanently, need help. Look for a mentor, somebody that you trust and you can talk with honestly. Speak your mind, you will find that most people are also feeling like an impostor or overwhelmed or do not know how to deal with some situations. Remember that we scientists are also humans. As humans, we have a lot of needs, do not forget to have fun, to go on vacation, to have babies and/or pets and/or plants and/or hobbies, to read novels that are not about science, to binge Netflix, to sleep late from time to time, to find the tribe you belong to, to get surrounded by good friends. And as humans scientists, be humble, kind, and respectful with everything and everyone and with yourself.

### 2.10 What has been your most rewarding experience so far? Why?

In 2014 I was honoured to give a plenary talk at ASV (American Society of Virology), and there I found myself in this big room full of people (1000? 2000?), and I had to convey this controversial message that in insects, RNA viruses are copying pieces of themselves into DNA. English has always been difficult for me, and my accent makes Shakespeare roll over in his grave. I always joke that I need subtitles when I speak. So here I am, from my native Cordoba to ASV and I am so scared and the “presenter view mode” of PowerPoint is not working, therefore I cannot read all the words I wrote in capitals to make the links between slides …. Somehow I delivered this talk, perhaps the most amazing talk I have ever give in my life, and the auditorium was captivated. But the after talk was the most rewarding experience. Two things struck me. The last night of the meeting, a group of students came to me with a peacock feather as a present for giving the most inspiring talk of all the meeting. They were now convinced they wanted to do a career in science. I have treasured the peacock feather in my office since then. This same night two girls from South America came to me and asked if they could hug me. They told me that during the talk, I showed them that it was possible to come from a disadvantaged environment and get far; I was a symbol of hope that they too they could succeed. I do not know who they were and where they are today, but I seriously hope that are rocking science somewhere in the world.

